# Single-cell RNA profiling of oligodendroglial lineage cells derived from iPSCs carrying Parkinson’s disease-relevant LRRK2-G2019S mutation

**DOI:** 10.1016/j.isci.2026.116368

**Published:** 2026-06-19

**Authors:** Nasser Karmali, Wiebke Kessler, Mohammad Dehestani, Wenhua Sun, Layla Drwesh, Polina Volos, Stanislav Tsitkov, Ashutosh Dhingra, Christian Thomas, Salvador Rodriguez-Nieto, Julia Tietz, David Schafflick, Noémia Fernandes, Julia Fitzgerald, Ernest Fraenkel, Thomas Gasser, Nisha Mohd Rafiq, Tanja Kuhlmann, Vikas Bansal

**Affiliations:** 1 German Center for Neurodegenerative Diseases (DZNE), 72076 Tübingen, Germany; 2 Interfaculty Institute of Biochemistry, University of Tübingen, 72076 Tübingen, Germany; 3 Institute of Neuropathology, University Hospital Münster, 48149 Münster, Germany; 4 Hertie Institute for Clinical Brain Research, University of Tübingen, 72076 Tübingen, Germany; 5 Graduate Training Centre of Neuroscience (GTC), University of Tübingen, 72076 Tübingen, Germany; 6 Department of Biological Engineering, Massachusetts Institute of Technology, Cambridge, MA 02139, USA; 7 Institute of Translational Neurology, University Hospital Münster, 48149 Münster, Germany; 8 Department of Psychiatry and Psychotherapy, Central Institute of Mental Health, Medical Faculty Mannheim, Heidelberg University, 68159 Mannheim, Germany

**Keywords:** Parkinson's disease, LRRK2, iPSCs, oligodendrocytes, oligodendrocyte precursor cells, scRNA-seq, cilia, semaphorin-plexin, hedgehog

## Abstract

Recent findings indicate oligodendrocytes (ODCs) and their progenitors are vulnerable in Parkinson’s disease (PD) pathogenesis. Notably, oligodendrocyte precursor cells (OPCs) exhibit high endogenous expression of the PD-relevant gene *LRRK2*. We differentiated iPSCs carrying the PD-relevant LRRK2 p.G2019S mutation into oligodendroglial lineages and performed single-cell RNA sequencing. Cell-type composition analysis revealed an increased proportion of OPCs, proliferating OPCs, and ciliated ependymal cells in LRRK2 lines, all of which are characterized by *LRRK2* expression. Differential expression analysis revealed transcriptomic changes in several pathways, including downregulation of genes related to myelin assembly in ODCs, semaphorin-plexin pathway in OPCs, and cilium movement in proliferating OPCs. Pseudotemporal trajectory analysis revealed that *SHH* had significantly altered expression along the pseudotime, accompanied by higher expression levels in LRRK2 lines. We provide a single-cell transcriptomic atlas of iPSC-derived oligodendroglial lineage cells carrying the PD-relevant LRRK2 p.G2019S mutation and control lines.

## Highlights

• Differentiated iPSCs carrying LRRK2 p.G2019S mutation into oligodendroglial lineages
• Single-cell RNA data from human iPSCs-derived cell types with or without the mutation
• OPCs and ODCs derived from iPSCs provide valuable cellular context for genetic risk

## Graphical abstract

**Figure:**
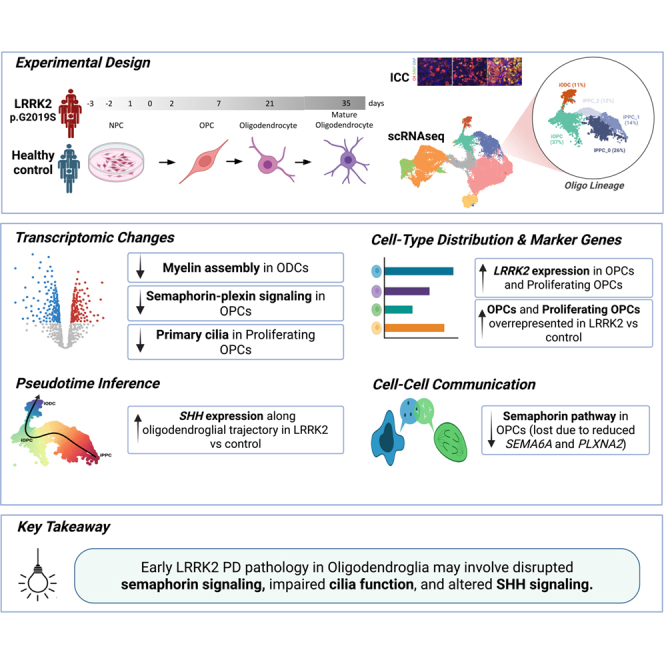

## Teaser

Molecular biology; Neuroscience; Cell biology; Bioinformatics; Omics

## Introduction

Parkinson’s disease (PD) is the second most common age-related neurodegenerative disorder characterized by the loss of dopaminergic neurons in the substantia nigra region of the brain.^1^ In fact, PD is the most common synucleinopathy to date that displays accumulation of aggregated form of α-synuclein protein encoded by *SNCA* gene. While the exact cause of PD remains elusive, genetic factors have been identified as significant contributors to the disease pathogenesis.^2^^,^^3^ Among the various genetic factors associated with PD, the LRRK2 p.G2019S point mutation that results in abnormally high kinase activity, is recognized as one of the most prevalent variants worldwide implicated in the familial form of PD. In Ashkenazi Jewish and North African Arab populations, significantly higher prevalence was found, occurring in up to 20%–40% of both familial and sporadic cases.^3^ The LRRK2 p.G2019S mutation is a missense mutation in the leucine-rich repeat kinase 2 (*LRRK2*) gene, which encodes a multifunctional protein involved in various cellular processes, including mitochondrial homeostasis, autophagy, and lysosomal function.^4^^,^^5^ Nearly two decades after linking *LRRK2* to PD,^6^^,^^7^ extensive research has been conducted to uncover the mechanistic pathways through which this mutation contributes to PD pathogenesis. The research particularly focuses on its effects on pathways such as mitophagy and lysosomal function in neurons, especially dopaminergic neurons.^8^^,^^9^

Advanced high-throughput single-cell omics analyses have identified a significant association between PD genetic risk and oligodendroglial lineage cells^10^^,^^11^^,^^12^^,^^13^ (including both oligodendrocyte (ODC) precursor cells and ODCs), indicating their potential role in the development of the PD. ODCs, the myelinating cells of the central nervous system, are derived from oligodendrocyte precursor cells (OPCs), and play a crucial role in maintaining the integrity and function of axons, facilitating efficient signal transmission between neurons.^14^^,^^15^^,^^16^ While traditionally recognized for their crucial role in myelination, ODCs and OPCs are now acknowledged for their wide and dynamic range of functions.^16^^,^^17^^,^^18^^,^^19^^,^^20^ ODCs not only form dynamic myelin sheaths around axons, which play a crucial role in higher brain functions such as learning and memory^21^^,^^22^ but also provide vital metabolic support to neurons,^15^ highlighting their contribution to overall brain function. OPCs are integrated into local neural circuits^23^ and participate in synaptic^24^ and axonal remodeling,^25^^,^^26^ phagocytosis process,^27^ and immunomodulation.^28^ Intriguingly, it has been observed that the *LRRK2* gene is expressed at significantly higher levels in OPCs compared to other cell types in the substantia nigra (SN).^12^^,^^29^^,^^30^ Hence, a clear avenue of investigation would involve exploring the effects of the LRRK2 p.G2019S mutation on oligodendroglial lineage cells and their potential role in PD development and progression.

**Figure 1:**
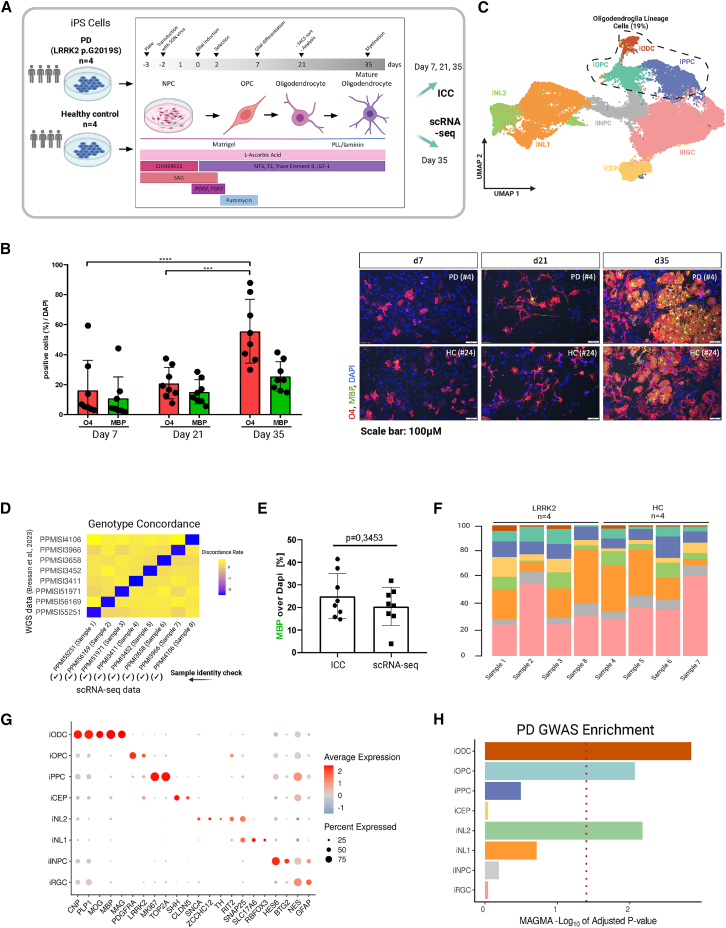
Overview of experimental plan, imaging, and scRNA-seq analysis (A) Schematic overview of the experimental plan. (B) Barplot representation of positive cells detected by immunocytochemistry (ICC) for oligodendrocyte markers O4 and MBP normalized to the total number of cells for day 7, 21, and 35 of the differentiation protocol. (∗∗∗∗ represents *p* value <0.001 and ∗∗∗ represents *p* value <0.01, one-way ANOVA). The right panel shows representative images of the PD and HC ICC staining. Red indicates O4^+^, green indicates MBP^+^ and blue indicates DAPI. (C) Uniform manifold approximation and projection (UMAP) visualization of the scRNA-seq clusters from 35,184 high quality cells. Cells are color-coded based on their assigned clusters; iPSC-induced oligodendrocytes (iODCs), iPSC-induced oligodendrocyte precursor cells (iOPCs), iPSC-induced proliferative precursor cells (iPPCs), iPSC-induced ciliated ependymal cells (iCEP), iPSC-induced neuron-like cells 2 (iNL2), iPSC-induced neuron-like cells 1 (iNL1), iPSC-induced intermediate neuronal progenitor cells (iINPC), and iPSC-induced radial glial cells (iRGCs). (D) Genotype concordance showing the consistency of genotypes between the scRNA-seq data and donor (blood-derived) whole-genome sequencing (WGS). (E) Percentage of MBP+ cells detected by ICC and scRNA-seq normalized to the total number of cells. Due to low *MBP* gene expression in sample 8, it was subsequently excluded from downstream analysis. P-value was calculated using a t-test. (F) Barplot representing the distribution of cell-type percentages across sample and mutation. (G) Dotplot illustrating the expression of known gene markers for cell identity. (H) Multi-marker analysis of genomic annotation (MAGMA) gene set enrichment based on 29,559 high quality cells showed significant associations with iPSC-induced oligodendrocytes (iODCs), iPSC-induced oligodendrocyte precursor cells (iOPCs) and iPSC-induced neuron-like 2 (iNL2) cells.

We aimed to create a first single-cell transcriptomic atlas of oligodendroglial lineage cells derived from induced pluripotent stem cells (iPSCs) by comparing LRRK2 p.G2019S PD-relevant mutation (hereafter referred to as PD lines) with healthy controls (HCs). By differentiating specific iPSC lines to an oligodendroglial lineage, we were able to explore the genetic effects of the LRRK2 p.G2019S mutation at an unprecedented level and created a comprehensive molecular cell atlas using single-cell transcriptomics coupled with immunocytochemistry (ICC) (Figure 1A). Using state-of-the-art computational analysis methods, we found significant dysregulation of the genes related to pathways like chemotaxis, calcium-mediated signaling, cell-cell adhesion, myelin assembly, fatty acid transport, I-kappaB kinase/NF-κB signaling, mitochondrion disassembly, semaphorin-plexin, sonic hedgehog (SHH), and cilia movement. We anticipate that our single-cell RNA sequencing (scRNA-seq) resource will aid in understanding the disease mechanisms and identifying potential therapeutic targets in PD, with a particular focus on oligodendroglial cells.

## Results

We used 8 iPSC lines, comprising 4 HCs and 4 with the LRRK2 p.G2019S mutation, which will be referred to as PD lines (Table S1). These lines were obtained from the Parkinson’s Progression Markers Initiative^31^ (PPMI; https://www.ppmi-info.org/) and were also used in our previous consortium study, the Foundational Data Initiative for Parkinson Disease^32^ (FOUNDIN-PD; https://www.foundinpd.org/). The iPSC lines were differentiated into oligodendroglial lineage cells using a previously published protocol,^33^ and their efficiency was evaluated through ICC at day 7, 21, and 35 (Figures 1A and 1B; Table S1). The O4^+^ cells (O4 antigen is a cell-surface sulfatide expressed on late-stage OPCs) were significantly increased from day 7 to day 35 and accounted for 55.6% ± 7.5%of DAPI^+^ cells at day 35 (Figure 1B). Deviating results from previously published study could be due to the different cultivation conditions of the neuronal precursor cells (NPCs).^34^^,^^35^^,^^36^ Next, we conducted scRNA-seq on differentiated iPSCs at day 35 (Table S1). Following data cleaning and quality control (see STAR Methods), we analyzed 35,184 high-quality single cells (18,125 cells from PD and 17,059 cells from HC). Clustering analysis revealed 8 major cell clusters identified as iPSC-induced oligodendrocytes (iODCs), iPSC-induced oligodendrocyte precursor cells (iOPCs), iPSC-induced proliferative precursor cells (iPPCs), iPSC-induced ciliated ependymal cells (iCEPs), iPSC-induced neuron-like cells 2 (iNL2), iPSC-induced neuron-like cells 1 (iNL1), iPSC-induced intermediate neuronal progenitor cells (iINPCs), and iPSC-induced radial glial cells (iRGCs) (Figure 1C). Approximately 74% of the total cells were identified as radial glial cells, neuron-like cells, and neuronal progenitors, while about 19% and 7% were oligodendroglial lineage cells (iODCs, iOPCs, and iPPCs) and ciliated ependymal cells, respectively (Figure 1C). The sample identities were verified by checking genotype concordance between previously published whole-genome sequencing (WGS) data^32^ and our generated scRNA-seq data (Figure 1D; Table S2). Subsequently, we compared the ICC-based estimation of myelin basic protein (MBP^+^ cells range: 15%–40%), a major constituent of the myelin sheath of ODCs, with the percentage of positive cells obtained from scRNA-seq data (range: 4%–32% and range: 16%–32% without sample 8) and found a similar distribution (Figure 1E), with a significant overall correlation coefficient (Spearman’s R = 0.69, Table S1). Of note, Sample 8 had the lowest percentage of MBP^+^ cells (4%) and the fewest reads (Table S1) as well as showed no contribution to the iODCs cell type (Figure 1F; Table S3) and was therefore excluded from the downstream analysis. Clusters were annotated based on the expression of known cell-type markers: iODC (marked by *CNP*, *PLP1*, *MOG*, *MAG*, and *MBP*), iOPC (marked by platelet-derived growth factor receptor alpha, *PDGFRA*), iPPC (marked by *PDGFRA*, *MKI67* and *TOP2A*), iCEP (marked by *SHH* and *CLDN5*), iNL2 (marked by *SNAP25*, *SNCA*, *TH* and *ZCCHC12*), iNL1 (marked by *SNAP25*, *SLC17A6*, and *RBFOX3)*, iINPC (marked by *HES6* and *BTG2*) and iRGC (marked by *NES* and *GFAP*) (Figure 1G; Table S4). Given that approximately 16% of cells within the iRGC cluster express *AQP4*, it is likely that this cluster includes a small subset of astrocyte-like cells. Notably, *LRRK2* demonstrated elevated expression levels in the iOPCs, iPPCs as well as in the iCEPs, consistent with earlier findings by Wang and colleagues,^37^ who observed high *LRRK2* expression in OPCs, microglia (cell type not present in our data) and endothelial cells from postmortem human substantia nigra single-nuclei RNA-seq data. Conversely, as reported by Wang and colleagues,^37^ *RIT2*, a PD susceptibility gene, was expressed not only in neuronal clusters but also in iOPCs. Integration of scRNA-seq data using Multi-marker Analysis of GenoMic Annotation (MAGMA_Celltyping) with PD genome-wide association studies (GWASs) showed a significant association (FDR-adjusted *p* < 0.05) of iODCs, iOPCs and iNL2 with PD-linked risk loci (Figure 1H; Table S5). These results provide a cellular context that is appropriate for modeling the genetic risk in PD.

**Figure 2:**
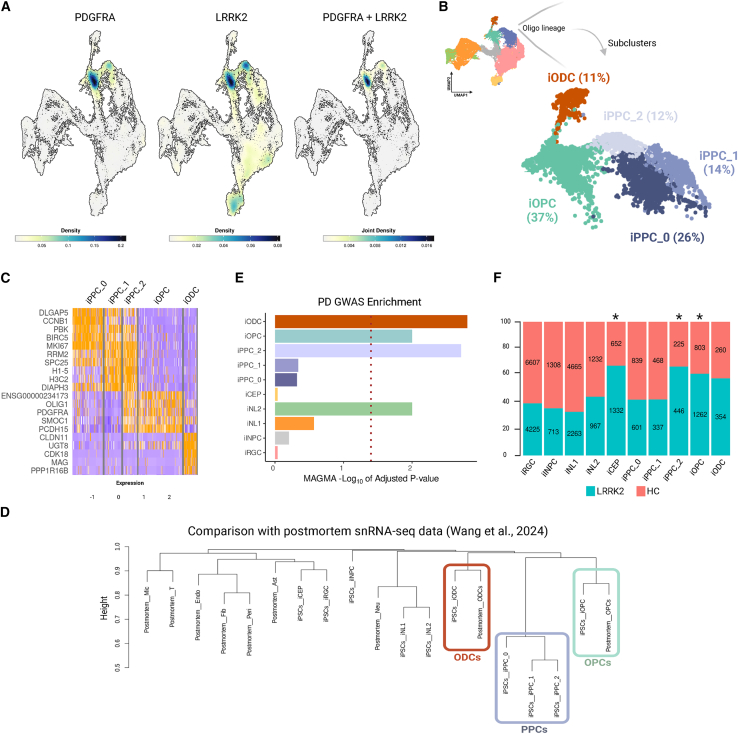
Sub-clustering of iPPC (A) Density plot representation of *PDGFRA* and *LRRK2* expression on Uniform manifold approximation and projection (UMAP) visualization of cell types. The dark blue color represents a high expression. (B) UMAP visualization of cells of oligodendroglial lineage with sub-clustering analysis of iPSC-induced proliferative progenitor cells (iPPC). Percentages were calculated based on the total number of oligodendroglial lineage cells (5,595 cells). (C) Heatmap of the top markers for the iPPC clusters, iOPCs, and iODCs. The dark yellow color represents a high expression. (D) Clustering of cell type identities in comparison with postmortem snRNA-seq data from Wang et al.^37^ (E) Multi-marker analysis of genomic annotation (MAGMA) gene set enrichment with iPPC clusters showed significant associations with iPSC-induced oligodendrocytes (iODCs), iPSC-induced oligodendrocyte precursor cells (iOPCs), iPSC-induced neuron-like 2 (iNL2) cells and iPSC-induced proliferative progenitor cell 2 (iPPC_2). (F) Distribution of cell-type proportion with speckle analysis for statistical testing for differences in cell type composition; (∗ represents *p* value <0.1). The red color represents healthy controls (HC) and blue represents the LRRK2 Parkinson’s disease (PD) samples.

The *PDGFRA* and *LRRK2* genes were expressed in both iOPCs and iPPCs (Figure 2A). However, while *LRRK2* expression was also widespread in progenitors and iCEPs, *PDGFRA* expression was more specifically restricted to iOPCs and iPPCs (Figure 2A). The sub-clustering of iPPCs revealed three subtypes: iPPC_0, iPPC_1, and iPPC_2 (Figure 2B). As expected, all three subtypes showed significant expression of a proliferating marker *MKI67*. *PDGFRA* was expressed only in iPPC_0 and iPPC_2, indicating that these are OPC-like cells in a cycling state (Figure 2C; Table S4). Next, we assessed the similarity between the marker gene signatures of our cultured oligodendroglial lineage cells and those from postmortem human substantia nigra single-nuclei RNA-seq data by Wang and colleagues^37^ (Figure 2D). Indeed, as anticipated, the iODCs clustered with human tissue ODCs, while the iOPCs clustered with human tissue OPCs (Figure 2D). This high concurrence between our iPSC-derived oligodendroglial cells and postmortem cells further strengthens the use of these cells as models. Apart from iODCs, iOPCs, and iNL2, which were previously associated with risk loci, MAGMA analysis following sub-clustering identified a significant association of iPPC_2 with PD GWAS (Figure 2E; Table S5). Then, we analyzed differences in cell type proportions using a robust method, *speckle*,^38^ that accommodates variation in biological replicates (see STAR Methods). We observed that all three cell types that exhibited significant *LRRK2* expression were enriched in PD lines compared to the controls, with *p* values of 0.03 for iPPC_2, 0.05 for iCEP, and 0.07 for iOPC (Figure 2F; Table S6).

**Figure 3:**
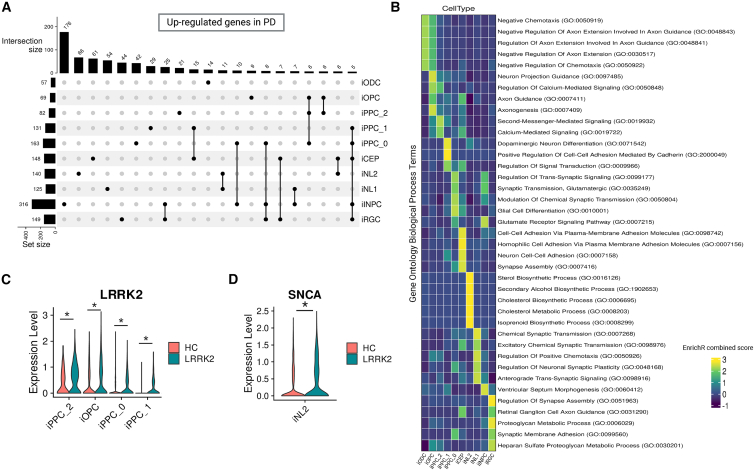
Differentially expressed upregulated genes in each cell-type (A) UpSet plot representing upregulated differentially expressed genes in each cell type and the number of intersections between cell types with equal or more than 5 DEGs in common. (B) Gene ontology enrichment analysis of upregulated genes. Top five biological process terms for each gene list indicated. EnrichR combined score (higher score shown in yellow and green) is calculated by the logarithmic transformation of the *p* value obtained from Fisher’s exact test, multiplied by the *Z* score representing the deviation from the expected rank. (C) Violin plot representing the gene expression level of *LRRK2* in HC and PD lines for the oligodendroglial lineage clusters. (D) Violin plots representing the gene expression level of *SNCA* in HC and PD lines for the iNL2 cluster (∗ represents adjusted *p* value <0.05).

**Figure 4:**
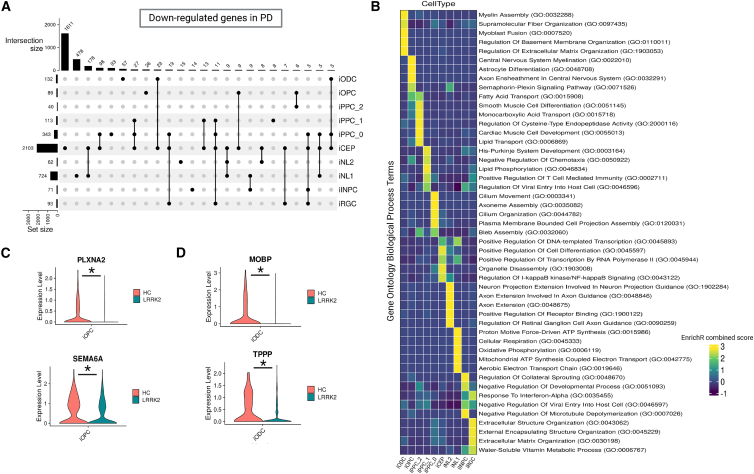
Differentially expressed downregulated genes in each cell-type (A) UpSet plot representing downregulated differentially expressed genes in each cell type and the number of intersections between cell types with equal or more than 5 DEGs in common. (B) Gene ontology enrichment analysis of downregulated genes. Top five biological process terms for each gene list indicated. EnrichR combined score (higher score shown in yellow and green) is calculated by the logarithmic transformation of the *p* value obtained from Fisher’s exact test, multiplied by the *Z* score representing the deviation from the expected rank. (C) Violin plots representing the gene expression level of *PLXNA2* and *SEMA6A* in HC and PD lines for the iOPC cluster. (D) Violin plots representing the gene expression level of *MOBP* and *TPPP* in HC and PD lines for the iODC cluster (∗ represents adjusted *p* value <0.05).

Differential gene expression analysis identified a total of 1,380 genes (795 unique genes) upregulated in PD cell lines (Figure 3; Table S7), whereas 3,770 genes (2,870 unique genes) were downregulated (Figure 4; Table S7). The significantly higher number of downregulated genes compared to upregulated ones was attributed to the iCEP and iNL1 clusters. Consequently, most upregulated genes were uniquely present in one cell type (Figure 3A), while most downregulated genes overlapped with iCEP or iNL1 (Figure 4A). Nevertheless, we observed a higher number of downregulated genes compared to upregulated genes in iODCs, iOPCs, and iPPC_0 (Figures 3A and 4A). Conversely, in the iPPC_1 and iPPC_2 clusters, there were fewer downregulated genes compared to upregulated genes. Gene Ontology (GO) analysis revealed that upregulated genes in iODCs and iOPCs were associated with negative chemotaxis and axon guidance related terms (Figure 3B; Table S8). Intriguingly, both iOPCs and iPPC_2 clusters displayed “Regulation of Calcium-Mediated Signaling” biological process term due to the upregulation of *RIT2*, *LRRK2*, and *PLCG2*. Following this clue, we found that *LRRK2* was significantly upregulated in iOPCs, iPPC_2 as well as iPPC_1 and iPPC_0 (Figure 3C). Notably, the alpha-synuclein gene (*SNCA)* was specifically upregulated in the iNL2 cluster, which was characterized by the expression of dopaminergic neuron markers such as *TH* and *ZCCHC12* (Figures 3D and 1G) and displayed cholesterol process related terms. On the other hand, genes associated with the “Semaphorin-Plexin Signaling Pathway” were downregulated in iNL2 (*NELL1* and *NELL2*) and iOPCs (*NELL2*, *SEMA6A*, and *PLXNA2*) (Figure 4B; Table S8). *SEMA6A* and *PLXNA2* were identified as specifically downregulated in iOPCs (Figure 4C). Additionally, terms related to fatty acid and lipid transport were enriched in iOPCs and iPPC_2 downregulated genes, while iPPC_1 showed enrichment of “Positive Regulation of T Cell-Mediated Immunity”. The downregulated genes in iPPC_0 cluster exhibited the distinct terms related to cilia movement. Finally, we found down-regulation of myelin assembly related genes in iODCs (Figure 4B; Tables S7 and S8). For instance, *TPPP*, a modulator of microtubule dynamics and essential for myelination, and *MOBP*, a key regulator of myelin assembly, were specifically downregulated in iODCs (Figure 4D).

**Figure 5:**
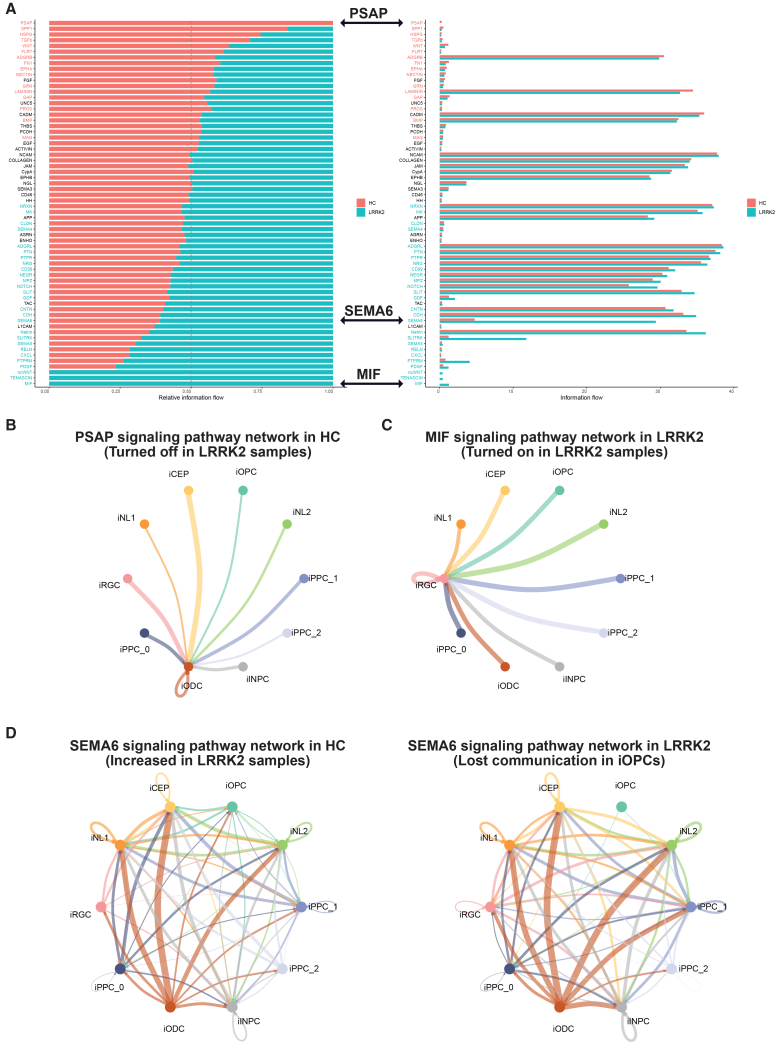
Cell-cell communication analysis (A) Table representing signals that contribute to outgoing and incoming signals for healthy controls (HC) and LRRK2 Parkinson’s disease (PD) lines. The red color represents HC and blue represents the LRRK2 PD samples. (B) The chord circle plot displays significantly interacting pathways and communication probabilities of PSAP signaling, which is present only in HC. (C and D) MIF signaling, which is present only in PD lines (D), and left: SEMA6 signaling in HC Right: SEMA6 signaling in PD lines.

Given the observed dysregulation in several signaling pathways, we hypothesized that this could result in significant signaling changes between cells across PD and HC lines. To investigate this, we used CellChat (v.2)^39^ to predict the main signaling inputs and outputs for cells under each condition and subsequently identified key signaling alterations between the PD and HC lines. We compared the information flow of each signaling pathway between PD and HC lines, defined as the sum of communication probabilities among all cell group pairs in the inferred network (Figure 5A; Table S9). Cell-cell communication analysis revealed significant changes in several signaling pathways, including the deactivation of PSAP signaling (Figure 5B) and the activation of migration inhibitor factor (MIF) signaling (Figure 5C) in PD lines. Notably, PSAP signaling was concentrated in iODCs, and *PSAP*, a lysosome-related gene, has been recently associated with PD risk.^40^ On the other hand, concerning macrophage MIF signaling (turned on in PD lines here), Park and colleagues^41^ recently demonstrated that the pathologic α-synuclein neurodegeneration in PD is mediated through MIF nuclease activity, and genetic depletion of MIF in mice prevents the loss of dopaminergic neurons. Additionally, we found the largest difference in information flow within the SEMA6 signaling network, which was overall increased in PD lines (Figure 5D; Table S9). However, iOPCs derived from these PD lines exhibited a significant reduction of information flow within the SEMA6 signaling pathway (Figure 5D, right).

**Figure 6:**
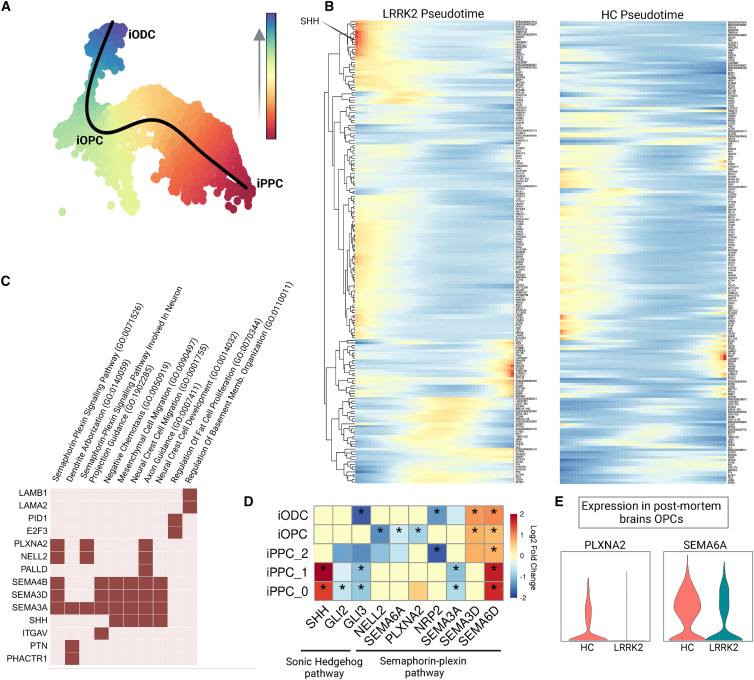
Single-cell trajectory and pseudotime analysis (A) Pseudotime analysis for the oligodendroglial lineage (cell types iODC, iOPC, and iPPC). The red color indicates lower and blue color indicates higher pseudotime, respectively. (B) Heatmap of genes overlapping between significant pseudotime-associated and condition test gene lists. The red color indicates higher expression. (C) Gene Ontology enrichment analysis of pseudotime-associated genes using biological process terms. (D) Heatmap showing differential expression of genes associated with semaphorin-plexin and sonic hedgehog pathways (∗ represents FDR-corrected *p* value <0.05). The semaphorin-plexin and sonic hedgehog related genes were extracted from CellChat signaling pathways using plotGeneExpression function. Additionally, GLI genes (*GLI1*, *GLI2*, and *GLI3*) were included. Only the genes that were significantly differentially expressed in at least one cell type are displayed. (E) Violin plots showing the expression of *SEMA6A* and *PLXNA2* in postmortem brain OPCs with and without LRRK2-G2019S mutation. The snRNA-seq data were reanalyzed from previous publication^13^ (GSE272760) using a bioinformatics pipeline from this study.

An unsupervised pseudotime analysis was conducted on oligodendroglial lineage cells, predicting a trajectory that spans from cycling cells of iPPCs to mature iODCs (Figure 6A). A total of 183 genes were identified that were significantly associated with pseudotime progression (FDR-adjusted *p* < 0.05) and also differentially expressed between conditions (FDR-adjusted *p* < 0.05) (Figure 6B; Table S10). GO analysis revealed that these genes were enriched for terms related to semaphorin-plexin signaling, negative chemotaxis, cell migration, and development (Figure 6C; Table S10). We noted increased expression of *SHH* at earlier pseudotime stages, along with higher expression levels in PD lines (Figure 6B), aligns with findings by Schmidt and colleagues,^42^ who reported that shortened primary cilia are associated with increased SHH signal transduction, and further demonstrated that inhibition of SHH signaling restored primary cilia morphology. We summarized the expression changes of genes related to the hedgehog and semaphorin-plexin signaling pathways in oligodendroglial lineage cells (Figure 6D). *SHH* and *SEMA6D* were upregulated in iPPC_1 and iPPC_0, whereas *GLI* genes and *SEMA3A* were downregulated. *SEMA6A* and *PLXNA2* were specifically downregulated in iOPCs. To validate our findings and extend our understanding of *SEMA6A* and *PLXNA2* expression in OPCs, we reanalyzed publicly available single-nuclei RNA sequencing data from human postmortem prefrontal cortex and anterior cingulate brain regions (Dehestani 2024). As presented in Figure 6E, *SEMA6A* and *PLXNA2* displayed a down-regulation in OPCs of PD brain samples carrying LRRK2 mutation compared to controls. Although both genes showed a down-regulation pattern, it is important to note that only *SEMA6A* reached statistical significance (log_2_ FC = −0.32, adjusted *p* = 1.46e−06).

**Figure 7:**
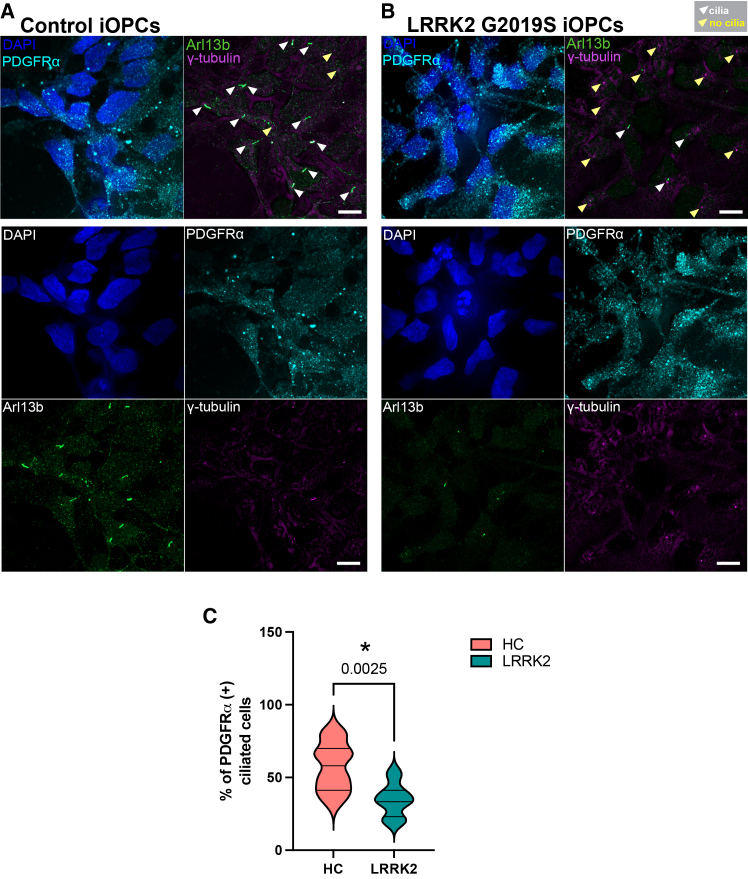
Reduced ciliation in LRRK2-G2019S iOPCs (A and B) Airyscan fluorescence images of control (A) and LRRK2 p.G2019S (B) iOPCs (day 11) immunolabeled with antibodies directed against OPC marker PDGFRα (cyan), ciliary marker Arl13b (green) and centrosomal marker γ-tubulin (green). DAPI was used to label the nuclei. Single channel images are shown beneath each merged panel (Scale bars, 10 μm). (C) Percentage of PDGFRα-positive cells with cilia (mean ± S.D.) from 6 mm^2^ areas on coverslips containing *n* ≥ 5 cells per area. Data were pooled from three independent control lines and three LRRK2 p.G2019S mutant lines. P-value was calculated using a Mann–Whitney test and was accepted as significant for p < 0.05.

Mutations in LRRK2 have previously been shown to cause primary cilia defects in striatal cholinergic interneurons and astrocytes.^43^ In line with this, we observed down-regulation of cilia genes in iPPC clusters (Figure 4B). Since *LRRK2* expression levels were found to be highly expressed in iOPCs (Figure 1G), we investigated whether the same LRRK2 mutation could play a role in OPC ciliation using three control (HC: #76, #97, #33) and three LRRK2-mutant lines (#2, #4, #12). At day 11, iOPCs were strongly positive for the ciliary marker Arl13b (Figure 7A), as shown in a representative image from HC line #33. Arl13b-positive cilia were clearly observed in cells co-stained with platelet-derived growth factor alpha (PDGFRα), a well-established OPC marker.^44^^,^^45^ This is in agreement with the previous report on the presence of primary cilia in OPCs.^46^ In contrast, the percentage of cilia-positive cells was lower in iOPCs harboring the LRRK2 p.G2019S mutation (Figures 7B and 7C)—with a representative image from line #4 illustrating this phenotype. Shortened cilia have previously been associated with higher *SHH* expression,^42^^,^^47^ which was also observed in our pseudotime analyses of oligodendroglial lineage in LRRK2 p.G2019S PD lines during early stages of oligodendroglial differentiation (Figure 6B), as cilia is absent in mature ODCs.^46^

## Discussion

In this study, we provide insights into the cellular and molecular mechanisms through which the LRRK2 p.G2019S mutation contributes to PD pathogenesis, with particular emphasis on oligodendroglial lineage cells. We used well-characterized PPMI human iPSC lines derived from PD-relevant LRRK2 p.G2019S mutation and from healthy controls, and generated scRNA-seq data from oligodendroglial lineage cells derived from these lines. We discovered significantly high expression of *LRRK2* in OPCs, proliferating OPCs as well as ependymal cell clusters compared to other cell types. All three cell types showed a significant increase in cell numbers in PD lines relative to controls, consistent with an influence of LRRK2 on cell proportions. The upregulation of *LRRK2* in PD OPCs and proliferating OPCs, along with *RIT2* (a PD risk gene^48^) and *PLCG2* (a phagocytosis- and immune-related gene^49^), consequently resulted in the enrichment of calcium-mediated signaling GO term. Previous studies have demonstrated that *LRRK2* overexpression increases autophagosome^50^ numbers via activation of a calcium-dependent pathway, potentially influencing the localization of α-synuclein.^51^ Since *LRRK2* is not expressed in neurons here as compared to OPCs, it is tempting to speculate that LRRK2 in OPCs may interact with SNCA in neurons by dysregulated calcium fluxes.

We identified dysregulated genes related to cilia movement, SHH signaling and semaphorin-plexin pathways in oligodendroglial lineage cells. Recent studies from various laboratories suggest that impairments in primary cilia and SHH signaling likely contribute to PD pathology.^42^^,^^47^^,^^52^^,^^53^^,^^54^^,^^55^ Importantly, the convergence on impaired ciliogenesis and SHH signaling in PD, incorporates data from both genetic and sporadic forms of the disease and is consistent across multiple tissues and iPS-derivation protocols. It has been proposed that *LRRK2* mutations affect the ability of cells to respond to cilia-dependent SHH signaling. Consistent with these observations, we identified a subcluster of proliferating OPCs that exhibited down-regulation of cilia-related genes, while *SHH* expression was significantly upregulated. Intriguingly, Pinskey and colleagues^56^ demonstrated that the GTPase-activating protein domain of plexin is necessary to enhance Hedgehog signaling at the level of GLI transcription factors, and this enhancement depends on intact primary cilia. Semaphorin-plexin pathways may represent a connection between primary cilia and SHH signaling. We observed a complex dysregulation of the genes in the semaphorin-plexin pathway: there was an overall increase in SEMA6 signaling communication in PD lines, yet OPCs derived from these lines specifically lost SEMA6 signaling. It is known that the migration of OPCs is modulated by a balance of effects mediated by members of the semaphorin and netrin families^57^ and Sema3A is known to be a potent, selective, and reversible inhibitor of OPC differentiation *in vitro*^58^*.* We found that *SEMA3A* and cilia-related genes are downregulated in the iPPC_0 cluster, which may contribute to cilia impairments in later-stage iOPCs. A recent study^46^ in the mouse primary visual cortex found that OPCs possess primary cilia. Whether human OPCs require intact primary cilia for semaphorin-plexin and SHH signaling is yet to be resolved. Restoring SEMA6A and PLXNA2 expression through transfection and assessing the resulting cellular phenotypes would provide direct evidence for their contribution to the observed transcriptional and cellular alterations. Another explanation for the overall increase in semaphorin-plexin-related genes and *SHH* is their involvement in cell migration,^59^^,^^60^ potentially as a response to the down-regulation of myelin assembly related genes in ODCs, with cilia impairments possibly hindering this migration. Alternatively, the specific down-regulation of *SEMA6A* in PD OPCs might delay ODC differentiation, leading to a reduced expression of myelin assembly related genes in ODCs^61^ from PD lines.

Altogether, we present the first scRNA-seq and immunocytochemistry data from oligodendroglial lineage cells derived from iPSCs carrying PD-relevant mutation LRRK2 p.G2019S mutation. It is tempting to speculate that semaphorin-plexin signaling, along with cilia movement and SHH signaling, might represent early events in PD pathology. The findings highlight the need for a deep exploration of the complex interactions among semaphorin-plexin, SHH, and cilium pathways in PD. Future studies involving other PD-relevant mutations, along with isogenic controls, will help elucidate the common and distinct pathways of PD pathoetiology in oligodendroglial cells. We envision that our work will serve as a valuable resource for uncovering potential targets in PD.

### Limitations of the study

Our study provides a single-cell transcriptomic atlas of iPSC-derived oligodendroglial lineage cells carrying the LRRK2 p.G2019S mutation; however, several important limitations should be considered when interpreting the findings. Critically, all differential expression, cell-cell communication, and trajectory analyses are based on transcriptional associations derived from scRNA-seq data. The conclusions drawn from these analyses might be correlative in nature, and establishing causal or mechanistic links will require future functional, perturbation-based, or *in vivo* experiments. In general, iPSC lines and differentiation protocols exhibit variability, and the transcriptional profiles of iPSC-derived oligodendroglial cells may not mirror those of mature or aged cell types. Additionally, RNA expression levels in scRNA-seq may be underestimated due to dropout effects. Moreover, RNA expression may not accurately reflect protein abundance because of post-transcriptional, translational, and post-translational regulatory mechanisms.

We conducted western blot analysis for LRRK2 (broadly upregulated in PD lines across multiple cell types), as well as SEMA6A and PLXNA2 (downregulated specifically in iOPCs). As expected, we observed a clear upregulation of LRRK2 at the protein level, consistent with its broad transcriptional increase in the scRNA-seq data (Figure S1). In contrast, changes in SEMA6A and PLXNA2 were not detectable by western blot, likely because their expression changes are restricted to a single cell type and thus diluted in bulk protein lysates. Future validation of protein-level changes in a cell type-specific manner such as examining myelin-related genes in ODCs, will be important to complement the transcriptional data and achieve the cellular resolution and lineage specificity provided by scRNA-seq.

Cell composition analysis using droplet-based scRNA-seq data can be sensitive to cell size, cell shape, and subsequent computational pipelines. For instance, ICC indicated that approximately 55% of cells were O4 positive (including ODCs with myelin sheaths), whereas scRNA-seq showed only 19% oligodendroglial lineage cells, emphasizing the importance of using ICC in conjunction with scRNA-seq. This discrepancy suggests the need for single-nuclei and/or full-length RNA sequencing. Furthermore, the mechanistic links proposed between ciliary dysfunction, semaphorin-plexin signaling, and PD pathogenesis remain to be functionally confirmed; experiments such as SHH inhibitor rescue assays and SEMA6A/PLXNA2 re-expression studies will be necessary to establish causality.

Finally, a larger cohort of iPSC lines with balanced sex and gender distribution, combined with multiple rounds of differentiation and comprehensive morphological and functional assessments, will be necessary to detect subtle molecular changes, to assess the influence of sex and better assess their disease relevance. We compared the iPSC-derived cell types generated in this study with those identified in postmortem brain samples and found transcriptional agreement in their identities. However, we cannot rule out the possibility that rare subpopulations of OPCs and ODCs cell types^62^^,^^63^^,^^64^ present in the aging human brain, and potentially vulnerable in PD, are underrepresented in our dataset. Nevertheless, iPSC technology remains a crucial platform for investigating PD pathogenesis and for preclinical evaluation of potential therapeutic treatments.^65^

## Resource availability

### Lead contact

Requests for further information and resources should be directed to the lead contact, Vikas Bansal (vikas.bansal@dzne.de).

### Materials availability

This study did not generate new unique reagents. iPSC lines were obtained from the Golub Capital iPSC Parkinson’s Progression Markers Initiative (PPMI) substudy (https://www.ppmi-info.org/access-data-specimens/request-cell-lines).

### Data and code availability

• Data: single-cell RNA-seq data are available at PPMI controlled-access repository with Project ID 338 and can be downloaded after approval by the PPMI Data Access Committee. Apply for access here https://www.ppmi-info.org/access-data-specimens/download-data => Genetic data. A Seurat object of the processed postmortem human brain snRNA-seq data has been uploaded to Zenodo https://doi.org/10.5281/zenodo.20200311
• Code: all original code has been deposited at Github (https://github.com/nnkarma12/scRNAseq_iOligo) and is publicly available as of the date of publication. DOIs are listed in the key resources table.
• Additional information: any additional information required to reanalyze the data reported in this paper is available from the lead contact upon request.

## Acknowledgments

Patient-derived iPSC lines, reprogrammed from the peripheral blood mononuclear cells, were obtained from the Golub Capital iPSC Parkinson’s Progression Markers Initiative (PPMI) substudy (https://www.ppmi-info.org/access-data-specimens/request-cell-lines) and were also used in FOUNDIN-PD consortium (https://www.foundinpd.org/). Data used in the preparation of this article were obtained (on 02-23-2024) from the PPMI database (https://www.ppmi-info.org/access-data-specimens/download-data), RRID: SCR_006431. For up-to-date information on the study, visit http://www.ppmi-info.org. PPMI—a public-private partnership—is funded by the 10.13039/100000864Michael J. Fox Foundation for Parkinson’s Research and funding partners, including 4D Pharma, Abbvie, AcureX, Allergan, Amathus Therapeutics, 10.13039/100018231Aligning Science Across Parkinson’s, AskBio, Avid Radiopharmaceuticals, BIAL, BioArctic, Biogen, Biohaven, BioLegend, BlueRock Therapeutics, Bristol-Myers Squibb, Calico Labs, Capsida Biotherapeutics, Celgene, Cerevel Therapeutics, Coave Therapeutics, DaCapo Brainscience, Denali, Edmond J. Safra Foundation, Eli Lilly, Gain Therapeutics, GE HealthCare, Genentech, GSK, Golub Capital, Handl Therapeutics, Insitro, Jazz Pharmaceuticals, 10.13039/100005565Johnson & Johnson Innovative Medicine, Lundbeck, Merck, Meso Scale Discovery, Mission Therapeutics, Neurocrine Biosciences, Neuron23, Neuropore, Pfizer, Piramal, Prevail Therapeutics, Roche, Sanofi, Servier, Sun Pharma Advanced Research Company, Takeda, Teva, UCB, Vanqua Bio, Verily, Voyager Therapeutics, the 10.13039/100019889Weston Family Foundation, and Yumanity Therapeutics. We would like to thank all of the participants who donated their time and biological samples to PPMI. We also thank Peter Heutink for initial support and discussions and Friederike Pfeiffer for Human MBP antibody. This work was funded in part by 10.13039/100000864The Michael J. Fox Foundation for Parkinson’s Research, MIT MISTI Global Seed Fund and a 10.13039/501100001659DFG grant (project number 513977564 to V.B.). V.B. is also supported by a Career Development Fellowship at 10.13039/501100005224DZNE Tuebingen. BioRender was used to prepare Figure 1A.

## Author contributions

V.B. conceived the project. J.F., E.F., T.G., N.M.R., T.K., and V.B. provided the resources, contributed in funding acquisition, and supervised the study. M.D., N.K., S.T., C.T., and V.B. performed the computational analysis of RNA-seq. T.K. conceived the oligodendroglia differentiation experiments. W.K. performed the oligodendrocyte differentiation experiments, staining, and image analysis. W.S. carried out the genotype check analysis. L.D. and J.F. performed western blot experiment and analysis. N.K., P.V., J.F., and N.M.R. performed the cilia stainings in iOPCs. A.D. and S.R.-N differentiated the iPSCs to NPCs. J.T., D.S., and N.F. performed cell isolation and 10x Genomics library preparation. M.D., N.K., and V.B. wrote the manuscript with input from all authors. All authors have read and approved the final version of the manuscript.

## Declaration of interests

The authors declare that they have no competing interests.

## STAR★Methods

### Key resources table

**Table:** 

REAGENT or RESOURCE	SOURCE	IDENTIFIER
**Antibodies**		

Mouse anti-O4-APC Antibody	Miltenyi Biotech	130-118-978; RRID:AB_2751598
Mouse anti-SEMA6A	Santa Cruz	sc-398302; RRID:AB_627960
Rabbit anti-Plexin-A2	Aviva Systems Biology	ARP49932_P050; RRID:AB_10642124
Rabbit anti-LRRK2	Abcam	ab133474; RRID:AB_2713963
Rat anti-MBP	Abcam	ab7349; RRID:AB_305584
Goat anti-PDGFRα	R&D Systems	AF-307-SP; RRID:AB_2089278
Rabbit anti-ARL13B	Proteintech	17711-1-AP; RRID:AB_10694363
Mouse anti-gamma tubulin	Proteintech	T6557; RRID:AB_477584
Alexa Fluor 488 Goat Anti-Rat IgG (H + L)	Jackson/Dianova	112-545-167; RRID:AB_2338362
Alexa Fluor 488 Anti-Mouse	Jackson/Dianova	115-545-166; RRID:AB_2338852
Cy3 Anti-Mouse IgG H + L	Jackson/Dianova	115-165-062; RRID:AB_2338685
Cy3 Anti-Rat IgG H + L	Jackson/Dianova	112-165-167; RRID:AB_2338251
Goat anti-rabbit HRP	ProteinSimple	042-206; RRID:AB_2860577
Goat anti-mouse HRP	ProteinSimple	042-205; RRID:AB_2860576
Goat IgG HRP	R&D Systems	HAF109; RRID:AB_355895
DAPI (FluorCare)	Carl Roth	ROTI®Mount FluorCare DAPI; RRID:AB_2650999

**Bacterial and virus strains**		

SON-Puro lentivirus	Ehrlich et al.^33^	N/A

**Chemicals, peptides, and recombinant proteins**		

Essential 8 Flex Medium	Thermo Fisher Scientific	A2858501
Matrigel hES-qualified	Corning	354277
Gentle Dissociation Reagent	StemCell Technologies	07174
Matrigel (NPCs)	Corning	354234
CHIR	R&D Systems	4423
PMA	Cayman Chemical	10009634
Ascorbic acid	Sigma-Aldrich	A8960
DMEM/F-12 GlutaMAX	Thermo Fisher Scientific	31331093
Neurobasal Medium	Thermo Fisher Scientific	21103049
*N*-2 Supplement	Thermo Fisher Scientific	17502048
B-27 Supplement minus Vit. A	Thermo Fisher Scientific	12587010
GlutaMAX	Thermo Fisher Scientific	35050
Insulin	Sigma-Aldrich	I9278
NEAA	Thermo Fisher Scientific	11140050
2-mercaptoethanol	Thermo Fisher Scientific	21985023
Penicillin/Streptomycin	N/A	N/A
Accutase	Sigma-Aldrich/Pan Biotech	A6964/P10-21100
Accutase	N/A	#P10-21100
CultrexTM coating solution	R&D Systems	3532-005-02
Puromycin	Gibco	A11138-03
BSA Fraction V	Gibco	15260037

**Critical commercial assays**		

10x Genomics Single Cell 3′ Kit v3.1	10x Genomics	PN-1000268
12–230 kDa separation module (8×25 capillary cartridge)	ProteinSimple	SM-FL004
66–440 kDa separation module (8×13 capillary cartridge)	ProteinSimple	SM-FL002
EZ standard pack I (fluorescent standards)	ProteinSimple	PS-ST01EZ-8/PS-ST03EZ-8
10x Sample Buffer	ProteinSimple	042–195
RePlex™ Reagent Kit	ProteinSimple	RP-001
Milk-free antibody diluent buffer 2	ProteinSimple	042–203
Secondary antibody module (includes ECL reagents)	ProteinSimple	DM-001
Total protein stain	ProteinSimple	DM-TP01
Tape Station High Sensitivity D5000/D1000 ScreenTape	Agilent	N/A
Bioanalyzer High-Sensitivity DNA Kit	Agilent	N/A
AO/PI Viability Assay	DeNovix	N/A

**Deposited data**		

Single-cell brain dataset	GEO	GSE272760 https://doi.org/10.5281/zenodo.20200311

**Experimental models: Cell lines**		

PPMI-derived iPSCs	PPMI/FOUNDIN-PD	https://www.ppmi-info.org/ https://www.foundinpd.org/

**Software and algorithms**		

Cell Ranger	10x Genomics	v8.0: RRID:SCR_017344
Seurat	Hao et al.^66^; https://github.com/satijalab/seurat	v5.0.3; RRID:SCR_016341
DoubletFinder	McGinnis et al.^67^; https://github.com/chris-mcginnis-ucsf/DoubletFinder	v2.0.3
Harmony	Korsunsky et al., 2019; https://github.com/immunogenomics/harmony	v1.2.0; RRID:SCR_022206
speckle R package	Phipson et al.^38^; https://github.com/Oshlack/speckle	v0.99.7; RRID:SCR_027464
ClusterMap	Gao et al.^68^; https://github.com/xgaoo/ClusterMap	v0.1.0
bcftools	https://samtools.github.io/bcftools	v1.20
CellChat	Jin et al.^39^; https://github.com/sqjin/CellChat	v2.1.2; RRID:SCR_021946
Slingshot	Street et al.^69^; https://github.com/kstreet13/slingshot	v2.10.0; RRID:SCR_017012
TradeSeq	Van den Berge et al.^68^; https://github.com/statOmics/tradeSeq	v1.16.0; RRID:SCR_019238
EnrichR	Xie et al.^70^; https://github.com/wjawaid/enrichR	RRID:SCR_001575
FIJI (ImageJ)	NIH	RRID:SCR_002285
GraphPad Prism	GraphPad Software	Version 10; RRID:SCR_002798

**Other**		

Olympus CKX53 Inverted Microscope	Olympus	RRID:SCR_025025
Chromium Controller iX	10x Genomics	RRID:SCR_024536
Zeiss LSM 980 Airyscan 2 microscope	Carl Zeiss Microscopy	RRID:SCR_025048

### Experimental model and study participants detail

#### Human iPSC lines

Eight human iPSC lines were used in this study: four carrying the LRRK2 p.G2019S mutation (LRRK2 lines: samples 1, 2, 3, 8; PPMI IDs: PPMI55251, PPMI56169, PPMI51971, PPMI4106) and four from healthy controls (HC: samples 4, 5, 6, 7; PPMI IDs: PPMI3411, PPMI3452, PPMI3658, PPMI3966). All lines were obtained from the Parkinson’s Progression Markers Initiative (PPMI) and were previously generated, characterized, and used in the FOUNDIN-PD consortium.^32^ Lines were derived from peripheral blood mononuclear cells of adult donors. Metadata are provided in Table S1 following the allowed access as given in the Table S1 of the FOUNDIN-PD consortium manuscript.^32^ Race, ethnicity, gender, sex and clinical details can be obtained from PPMI upon request and after approval by the PPMI Data Access Committee. Of the eight donors, two were female (one in each group) and six were male. This study was not powered to assess sex-based differences in the results, and this has been noted as a limitation. Participants provided written informed consent to be part of the PPMI (https://www.ppmi-info.org/). The differentiation of cell lines to Oligodendrocytes used in this study was approved by the local ethical committees in Münster (AZ 2016-026-f-S).

### Method details

#### Culturing of induced pluripotent stem cells (iPSCs)

The Parkinson’s Progression Markers Initiative (PPMI) iPSCs included in this study have previously been generated and characterized.^32^ Human iPSCs were cultured as colonies in feeder-free conditions using Essential 8 Flex Medium (Thermo Fisher Scientific, A2858501) on Matrigel hES-qualified (Corning, 354277) coated plates. Colonies were passaged as aggregates every 5–6 days. Briefly, iPSC colonies were treated with Gentle Dissociation Reagent (GDR, StemCell technologies, 07174) for 7 min at room temperature. GDR was then replaced with E−8 flex media and colonies were gently triturated by pipetting three times to obtain a suspension of small aggregates. Small colonies were plated onto Matrigel-coated plates, and the media was refreshed every other day.

#### Differentiation of iPSCs into neural progenitor cells (NPCs)

The small molecules NPCs (smNPCs) were derived from iPS cells using the method described in Reinhardt et al. 2013,^35^ applying a few adjustments as described in Dhingra et al., 2020.^71^ The smNPCs were cultured on Matrigel (Corning, 354234) coated plates in the expansion medium (N2B27) supplemented with 3 μM CHIR (R&D System, 4423), 0.5 μM PMA (Cayman Chemical, 10009634) and 64 mg/L Ascorbic acid (AA, Sigma-Aldrich, A8960) for up to 8 passages before use in any downstream experiments. N2B27 medium consisted of DMEM/F-12-GlutaMAX (Thermo Fisher Scientific, 31331093) and Neurobasal (Thermo Fisher Scientific, 21103049) mixed at 1:1 ratio, supplemented with 1:200 *N*-2 supplements (Thermo Fisher Scientific, 17502048), 1:100 B-27 supplements without vitamin A (Thermo Fisher Scientific, 12587010), 1:200 GlutaMAX (Thermo Fisher Scientific, 35050), 5 μg/mL insulin (Sigma-Aldrich, I9278), 1:200 non-essential amino acids (NEAA, Thermo Fisher Scientific, 11140050), 55 μM 2-mercaptoethanol (Thermo Fisher Scientific, 21985023), and 1:100 penicillin/streptomycin. The smNPCs were routinely passaged using Accutase (Sigma-Aldrich, A6964).

#### Differentiation of smNPCs into oligodendroglial-lineage cells

For the differentiation of small molecule neuronal precursor cell (smNPCs) into viral induced oligodendrocytes (hiOL), 80–90% confluent cells were detached with accutase (Pan Biotech, #P10-21100) and seeded in smNPC medium in CultrexTM (R&D systems #3532-005-02) -coated 6-well plates with a density of 3∗105 cells per well. The next day, cells were transduced with the SON-Puro lentivirus and were incubated overnight before washing on the next day. A day after washing, the differentiation was started by changing the medium to glial induction medium (GIM). For selection 2 μg/mL puromycin (Gibco, #A11138-03) was added from differentiation day 2 to day 7. Medium was changed to glial differentiation medium on day 4 of the differentiation and was changed every other day. To obtain a pure culture cells were FACS sorted on day 21 of differentiation for O4-APC (#130-118-978, Miltenyi Biotech). After the FACS sort cells were cultivated in GDM for another 14 days and medium was changed every other day. At day 35 of the differentiation cells were fixated with 4%PFA.

#### Immunocytochemistry (ICC)

For ICC, cells were cultured on CultrexTM (R&D systems #3532-005-02) -coated 48-well or 96-well plates. After fixation with 4% PFA for 20 min at RT, cells were washed twice with PBS and either stored at 4 °C or used immediately for staining. For blocking of non-specific binding sites, cells were incubated 45 min in blocking buffer, containing PBS with 5% normal goat serum and 5% fetal bovine serum (FBS), at RT. For intracellular binding epitopes, cells were subsequently permeabilized with 0.5% Triton X-100 in PBS for 5–7 min at RT. Afterward, primary antibodies (Table S11) were diluted in the blocking buffer and were applied. Cells were incubated at 4 °C overnight. The next day, cells were washed twice with PBS, Cy-3 or Alexa 488 conjugated antibodies (Table S11) were applied and incubated for 1 h at RT in the dark. For double stainings steps for applying primary and secondary antibodies were repeated. After washing cells twice with PBS, to counterstain the nuclei 1 μg/mL 4’-,6-diamidino-2-phenylindole (DAPI) in PBS was added for 10 min followed by additional three washing steps. To visualize fluorescent staining cells were analyzed by an Olympus CKX53 inverted microscope (Olympus). For each cell line, 10 images of a technical triplicate were taken and the mean value determined.

#### Fluorescence associated cell sorting (FACS)

To prepare cells for the FACS procedure, cells were detached with accutase (Pan Biotech, #P10-21100) 10 min at 37 °C. Afterward cells were resuspended and diluted in a ratio of 1:10 in split medium and centrifuged for 5 min at 200 rcf. After centrifugation, cells were resuspended in FACS buffer, which consists of PBS with 0.5% BSA Fraction V (Gibco, #15260037). Cell numbers were determined using a Neubauer counting chamber. Cells were stained with the O4-APC antibody (Miltenyi Biotech, #130-118-978) according to the manufacturer’s manuscript. For FACS sorting, cells were stored in ice.

#### Single-cell transcriptome library preparation and sequencing

Single-cell suspension concentration was determined by automatic cell counting (DeNovix CellDrop, DE, USA) using an AO/PI viability assay (DeNovix, DE, USA) and counting cells as dead cells. Single-cell gene expression libraries were generated using the 10x Chromium Next gel beads-in-emulsion (GEM) Single Cell 3′ Reagent Kit v3.1 (10x Genomics, CA, USA) according to manufacturer’s instructions. In brief, cells were loaded on the Chromium Next GEM Chip G, which was subsequently run on the Chromium Controller iX (10x Genomics, CA, USA) to partition cells into GEMs. Cell lysis and reverse transcription of poly-adenylated mRNA occurred within the GEMs and resulted in cDNA with GEM-specific barcodes and transcript-specific unique molecular identifiers (UMIs). After breaking the emulsion, cDNA was amplified by PCR, enzymatically fragmented, end-repaired, extended with 3′ A-overhangs, and ligated to adapters. cDNA QC and quantification was done with Tape Station High Sensitivity D5000 ScreenTape (Agilent, CA, USA). P5 and P7 sequences, as well as sample indices (Chromium Library Construction Kit, 10x Genomics, CA, USA) with Dual Index Kit TT Set A, (10x Genomics, CA, USA), were added during the final PCR amplification step. The fragment size of the final libraries was determined using the Bioanalyzer High-Sensitivity DNA Kit (Agilent, CA, USA). Library concentration of the final libraries was determined using the Tape Station High Sensitivity D1000 ScreenTape (Agilent, CA, USA). Single-cell RNA libraries were pooled and paired-end-sequenced on the Illumina NovaSeq 6000 platform (Illumina, CA, USA), using an S1 flow cell.

#### Single-cell RNA-seq quality control and analysis

The sequencing reads were demultiplexed using the 10x Genomics Cell Ranger v8.0 and to perform alignment against the 10x Genomics pre-built Cell Ranger reference GRCh38-2024-A to generate count matrices for each sample. Seurat^66^ objects v5.0.3 were created for each sample with a cut-off value of 200 unique molecular identifiers (UMIs) expressed in at least 3 cells. Cells with fewer than 1000 genes detected or more than 5% of reads mapping to mitochondrial genes were removed. Each sample was processed individually, and doublets and multiplets were filtered out using DoubletFinder v2.0.3^67^ with recommended settings. Following the filtering steps applied, the dataset contained 35184 high-quality cells that were used for the following analyses. The individual Seurat objects were merged and normalized by the SCTransform^72^ method in Seurat v5.0.3 with mitochondrial reads regressed out. Principal component analysis (PCA) was then performed on the normalized data using the ‘RunPCA’ function. Integration and batch correction across samples were achieved using the Harmony R package version 1.2.0 with the ‘HarmonyIntegration’ function. The first 30 principal components (PCs) from the Harmony-corrected data were used as inputs for downstream analyses. A shared-nearest-neighbor graph was constructed using the ‘FindNeighbors’ function and cells were clustered using the Louvain algorithm implemented in the ‘FindClusters’ function with a resolution of 0.1. Cluster 2 was then further sub-clustered using ‘FindSubCluster’ in order to separate the iOPC cluster and the iINPC cluster using the Louvain algorithm with a resolution of 0.1. The cell identities were saved in the metadata in the column ‘BroadCellType’ (Table S3). Sub-clustering was performed on the iPPC cluster using the Louvain algorithm with a resolution of 0.15 using the ‘FindSubCluster’ function. The sub-clustered cells were assigned new identities representing different iPPC cell types, and the metadata was updated accordingly under the column ‘CellType’. UMAP was performed to visualize the clusters in two dimensions using the ‘RunUMAP’ function. The Seurat function ‘FindAllMarkers’ with default parameters was used in order to identify markers for the clusters. Known markers based on previously reported literature were confirmed in order to determine the identity of each cluster (Table S4). The proportion of cell types was analyzed using the ‘speckle^38^’ R package v0.99.7 and the ‘propeller’ function with the ‘CellType’, ‘SampleID’ and ‘Mutation’ columns from the Seurat metadata as input (Table S6). To compare our data with postmortem brain tissues, ClusterMap^68^ v0.1.0 was executed on the top 500 genes (sorted by *p*-value) from each cluster with at least one log fold-change, resulting in a total of 5,000 marker genes from this study and 3,289 marker genes from Wang et al.^37^

#### Genotype and sample identity check

We evaluated the correlation between single-cell sequencing data and donor (blood-derived) whole-genome sequencing (WGS) data to validate sample origin and identity in our scRNA-seq data. The subset-bam v1.1.0 was used to subset BAM files by extracting the alignment records with valid filtered cell barcodes generated by Cell Ranger (https://github.com/10XGenomics/subset-bam). The cellsnp-lite^73^ v1.2.3 tool in Mode 2b was used in pseudo-bulk manner to call genotypes in scRNA-seq BAMs using the WGS VCF file from chromosome 12. The resulting VCF files from scRNA-seq data were indexed using bcftools^74^ v1.20. Following the indexing, sample concordance between the WGS VCF and single-cell VCF files was checked using the ‘gtcheck’ function in bcftools. Finally, a summary table was created with the discordant rates. The discordance rate indicates the level of inconsistency between the samples, where a lower discordance rate signifies higher consistency between the WGS and single-cell data (Table S2).

#### Differential expression and gene ontology enrichment analysis

Differential gene expression analysis was performed using the non-parametric Wilcoxon rank-sum test implemented in the ‘FindMarkers’ function of Seurat v5.0.3 with default parameters with the exception of the parameters ‘min.pct’ and ‘min.diff.pct’ where a threshold of 0.1 was used. Differentially expressed genes between PD and HC were determined for each cell type. Gene ontology enrichment analysis for biological processes was performed using EnrichR^70^ library GO_Biological_Processes_2023. Enrichr combined score is calculated by the logarithmic transformation of the *p*-value obtained from Fisher’s exact test, multiplied by the *Z* score representing the deviation from the expected rank (Table S7; DEGs; Table S8; GO).

#### Cell-type association with genetic risk of PD

Association analysis of cell type-specific expressed genes with genetic risk of PD was performed as described previously,^32^ using Multi-marker Analysis of GenoMic Annotation (MAGMA) v2.0.2, in order to identify disease-relevant cell types in the data.^75^^,^^76^ MAGMA, as a gene set enrichment analysis method, tests the joint association of all SNPs in a gene with the phenotype, while accounting for LD structure between SNPs. Competitive gene set analysis was performed on SNP *p*-values from the latest PD GWAS summary statistics including 23andMe data and the publicly available European subset of 1000 Genomes Phase 3 was used as a reference panel to estimate LD between SNPs. SNPs were mapped to genes using NCBI GRCh37 build (annotation release 105). Gene boundaries were defined as the transcribed region of each gene. An extended window of 10 kb upstream and 1.5 kb downstream of each gene was added to the gene boundaries.

#### Cell-cell communication analysis

We used CellChat^39^ v2.1.2 to perform a comparison analysis between LRRK2 and HC lines. As indicated by the vignette, we ran CellChat on each group (LRRK2 or HC) independently, and then merged the two CellChat objects. We used the functions computeCommunProb, filterCommunication (50 cells), computeCommunProbPathway, aggregateNet, and netAnalysis_computeCentrality with default parameters, utilizing CellChatDB.human as the ligand-receptor interaction database. The CellChat objects from LRRK2 and HC were merged using mergeCellChat function. To create Figure 5A and Table S9, the signaling networks were ranked based on the information flow using the rankNet function. We utilized the netVisual_aggregate function to visually compare cell-cell communication using a Circle plot as shown in Figures 5B and 5C.

#### Single-cell trajectory and pseudotime analysis

For the pseudotime analysis and trajectory inference, we utilized Slingshot^69^ v2.10.0. Our input data comprised a gene expression matrix extracted from a subset of fully processed Seurat object from previous steps, only including oligodendroglial lineage cells: iPPC_0, iPPC_1, iPPC_2, iOPC, and iODC. Slingshot operates by constructing a minimum spanning tree (MST) and subsequently fitting principal curves to the high-dimensional gene expression data. This approach effectively captures the underlying nonlinear relationships and delineates the structure of cellular differentiation and transitional trajectories. Importantly, it enables the inference of continuous cell progression through various developmental stages without the need to determine root cells explicitly (Figure 6A). Following trajectory inference, we used TradeSeq^77^ v1.16.0 to find pseudotime-associated genes which are differentially expressed between conditions. It fitted a generalized additive model to the expression data of each gene along the pseudotime. GAMs are flexible models that can capture complex, non-linear relationships between gene expression and pseudotime. AssociationTest implemented in this tool was used to firstly identify pseudotime-associated genes. Subsequently, its conditionTest function was executed to find genes that are differentially expressed between conditions. We proceeded to look at the overlaps (*n* = 183) among significant genes based on multiple testing adjusted *p*-values (<0.05) derived from both associated and differentially expressed gene lists. This analysis underscored distinct expression patterns among pseudotime-associated genes in LRRK2 and HC lines (Figure 6B). Finally, we utilized EnrichR to investigate the functional enrichment of these overlapping genes within biological processes. The semaphorin-plexin and sonic hedgehog related genes were extracted from CellChat signaling pathways using plotGeneExpression function. Additionally, GLI genes (*GLI1, GLI2, GLI3*) were included.

#### Immunofluorescence and fluorescence microscopy for cilia

For cilia stainings, three control (HC: #76, #97, #33) and three LRRK2-mutant lines (#2, #4, #12) were selected to differentiate (day 11) on glass slides. Cells were seeded on glass coverslips and fixed with 4% (v/v) paraformaldehyde in 1x phosphate-buffered saline (PBS) for 20 min followed by three washes in PBS. Cells were permeabilized with 0.5% (v/v) Triton X-100 in PBS for 10 min followed by three washes in PBS. They were further blocked for 30 min in 5% bovine serum albumin (BSA, Sigma-Aldrich) in PBS and then incubated overnight at 4 °C with the primary antibodies listed in Table S11. Subsequently, cells were washed with PBS thrice the following day and incubated with Alexa Fluor-conjugated secondary antibodies (Thermo Fisher Scientific) for 1 h at room temperature, followed by three washes in PBS. Coverslips were mounted on glass slides with ROTIMount FluorCare DAPI (Roth Carl) at 4°C. For imaging, The LSM 980 inverted confocal laser scanning microscope with Airyscan 2 (Carl Zeiss Microscopy) accompanied with 63×/1.40 numerical aperture (NA) plan-apochromat differential interference contrast (DIC) M27-Oil immersion objective and 32-channel gallium arsenide phosphide (GaAsP)-photomultiplier tubes (PMTs) area detector with 405 nm, 488 nm, 561 nm and 633 laser lines was used in this study. Images were acquired and processed using ZEN black imaging software (Zeiss).

#### Image and statistical analysis

Images were pseudocolor-coded, adjusted for brightness and contrast, using the open-source image processing software FIJI (ImageJ).^78^ For ciliary analyses, Mann–Whitney test was used when comparing two datasets. Differences were accepted as significant for *p* < 0.05. Prism version 10 (GraphPad Software) was used to plot, analyze, and represent the data.

#### Automated western blotting

Whole cell lysate (WCL) samples of iOPCs at day 35 of differentiation were analyzed using the JESS Simple WesternTM system (ProteinSimple, Bio-Techne) with the 12–230 kDa separation module (8 x 25 capillary cartridge, #SM-FL004) for detecting SEMA6A. And with 66–440 kDa separation module (8 x 13 capillary cartridge, #SM-FL002) for detecting LRRK2 and Plexin-A2. DTT, biotinylated molecular weight ladder, and fluorescent standards (EZ standard pack I #PS-ST01EZ-8 and #PS-ST03EZ-8) were reconstituted in double-distilled water (ddH2O) according to the manufacturer’s instructions. Lysates were mixed with 1x fluorescent master mix and diluted to a final protein concentration of 0.2–0.4 mg/mL using 0.1x sample buffer (from 10x stock solution, #042–195). Samples were denatured at 95 °C for 5 min before loading. A total of 4 μL (containing 0.8–1.6 μg protein) was loaded per well. The following antibodies with the indicated dilutions were used for analyzing the lysates: SEMA6A (#sc-398302 B-3, Santa Cruz) at 1:10, Plexin-A2 (ARP49932_P050) at 1:10, and LRRK2 (ab133474, Abcam) at 1:50. Samples were decorated with the antibodies in one probe and total protein stain (#DM-TP01) in the second probe using sequential detection by RePlexTM reagent kit (#RP-001). Samples were decorated with ready to use goat anti-rabbit secondary HRP-conjugated antibody (#042–206), with ready to use goat anti-mouse secondary HRP-conjugated antibody (#042–205) or with goat igG HRP-conjugated secondary antibody (HAF109, R&D) at 1:50. For preparing the final primary antibody dilution mix, milk-free antibody diluent buffer 2 was used (#042–203). Protein signal was developed using enhanced chemiluminescence (ECL) reagents provided in the secondary antibody module (#DM-001) and according to the manufacturer’s instructions. Data acquisition and analysis were performed using Compass for Simple Western (SW) software (ProteinSimple, Bio-Techne). Signal quantification was based on the area under the curve (AUC) of electropherogram peaks from the high dynamic range 4.0 generated for each target protein. For normalization, total protein staining was used as a loading control. The system’s in-capillary total protein detection module labeled all proteins in the separation matrix, allowing lane-by-lane normalization. Target protein signals were normalized to the total protein signal from the same capillary to account for variations in sample loading. Normalized quantification data were exported from Compass software to GraphPad (GraphPad Prism 10 Software) for statistical analysis and figure generation.

### Quantification and statistical analysis

Statistical details for all analyses are reported in the figure legends and main text. For immunocytochemistry quantification, a one-way ANOVA was used to assess differences in O4+ and MBP+ cell proportions across differentiation time points (day 7, 21, and 35). Spearman’s rank correlation coefficient was used to assess concordance between ICC-based and scRNA-seq-based MBP+ cell proportions across samples (R = 0.69, Table S1). For cell type proportion analysis, the propeller function in the speckle R package v0.99.7 was used, which applies a logit transformation to stabilize variance across biological replicates; *n* = 7 samples (3 HC and 4 PD) following exclusion of sample 8. Differential gene expression between PD and HC lines was assessed using the non-parametric Wilcoxon rank-sum test implemented in the FindMarkers function of Seurat v5.0.3; genes were considered differentially expressed at FDR-adjusted *p* < 0.05 with a minimum expression threshold of 0.1 (min.pct and min.diff.pct). Cell-type association with PD genetic risk was assessed using MAGMA v2.0.2 competitive gene set analysis on SNP *p*-values from the latest PD GWAS summary statistics; cell types were considered significantly associated at FDR-adjusted *p* < 0.05. Cell-cell communication analysis was performed using CellChat v2.1.2; signaling pathways were ranked by information flow defined as the sum of communication probabilities across all cell group pairs. For pseudotime-associated gene analysis, generalized additive models were fitted using TradeSeq v1.16.0; genes were considered significant at FDR-adjusted *p* < 0.05 for both the associationTest and conditionTest, and overlapping significant genes (*n* = 183) were taken forward for GO enrichment analysis. Gene ontology enrichment analysis was performed using EnrichR with the GO_Biological_Processes_2023 library. For ciliary analysis, a Mann-Whitney test was used to compare the percentage of PDGFRα-positive ciliated cells between HC and LRRK2 p.G2019S lines and differences were considered significant at *p* < 0.05. For western blot quantification, signal intensity was calculated as the area under the curve of electropherogram peaks using Compass for Simple Western software, normalized to total protein staining per capillary.

## Supplemental information

Document S1. Figure S1

Table S1. Sample overview with meta-data, related to Figure 1

Table S2. Genotype check for sample concordance and identity, related to Figure 1

Table S3. Number of cells across broad cell types and mutation group, related to Figure 1

Table S4. Known and identified cluster markers, related to Figure 1–2

Table S5. MAGMA scRNA-seq cell-type enrichment results, related to Figure 1–2

Table S6. Number of cells across cell types and mutation group with speckle analysis, related to Figure 1–2

Table S7. Differentially expressed genes between LRRK2 and HC in each cell type, related to Figure 3–4

Table S8. Enriched gene ontology terms for DEGs for all subclusters from EnrichR, related to Figure 3–4

Table S9. Cell-cell communication information flow statistics, related to Figure 5

Table S10. Trajectory-based genes associated with pseudotime and differential expression, related to Figure 6

Table S11. Description of antibodies for ICC and FACS, related to Figure 7
