# Supplementary material for: Single-cell RNA profiling of oligodendroglial lineage cells derived from iPSCs carrying Parkinson’s disease-relevant LRRK2-G2019S mutation
Source: iScience. 2026 Jun 19;29(7):116368. doi: 10.1016/j.isci.2026.116368 (PMC13315888; doi:10.1016/j.isci.2026.116368)
Supplement: Document S1. Figure S1 [file mmc1.pdf]

iScience, Volume *29*

Supplemental information

Single-cell RNA profiling of oligodendroglial lineage cells derived from iPSCs carrying Parkinson's disease-relevant LRRK2-G2019S mutation

Nasser Karmali, Wiebke Kessler, Mohammad Dehestani, Wenhua Sun, Layla Drwesh, Polina Volos, Stanislav Tsitkov, Ashutosh Dhingra, Christian Thomas, Salvador Rodriguez-Nieto, Julia Tietz, David Schafflick, Noémia Fernandes, Julia Fitzgerald, Ernest Fraenkel, Thomas Gasser, Nisha Mohd Rafiq, Tanja Kuhlmann, and Vikas Bansal

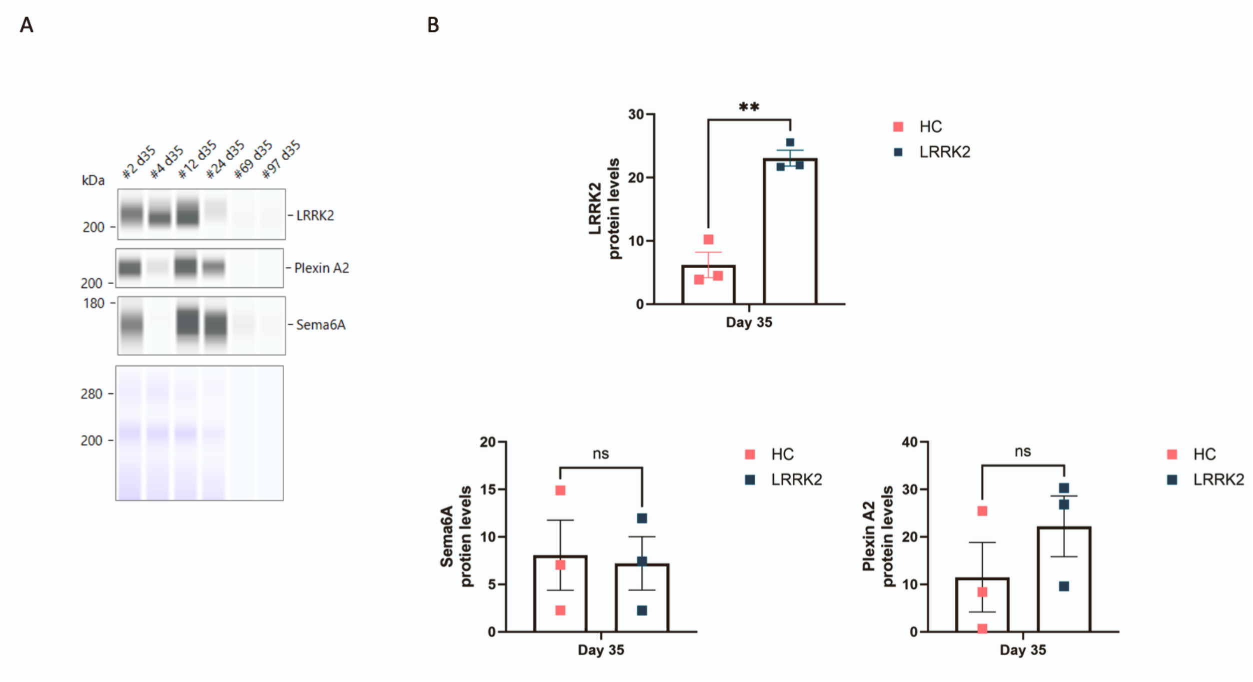

Figure S1: Western blot analysis of protein expression at day 35 of iPSCs differentiation into oligodendroglial lineage cells. (A) Whole cell lysates (WCL) from iPSC-induced oligodendrocytes at day 35 of differentiation, derived from healthy controls (#24, #76 (named #69), #97) and LRRK2 G2019S PD lines (#2, #4, #12), were analyzed using the Simple Western system (JESS, Bio-Techne). Lysates were probed with antibodies against LRRK2, Plexin-A2, and Sema6A. **(B)** Protein signal intensities were quantified using Compass for Simple Western (CompassForSW) software and normalized to total protein staining within each lane. Bar plots represent the average protein expression for each group; individual data points are shown as dots. Error bars indicate the standard error of the mean (SEM). Statistical significance between different groups was assessed using Welch's t-test with significance denoted by asterisks. Significance levels are indicated as follows: ns (not significant, $p > 0.05$), * ($p < 0.05$), ** ($p < 0.01$).
